# Searching for FHB Resistances in Bread Wheat: Susceptibility at the Crossroad

**DOI:** 10.3389/fpls.2020.00731

**Published:** 2020-06-11

**Authors:** Francis Fabre, Florian Rocher, Tarek Alouane, Thierry Langin, Ludovic Bonhomme

**Affiliations:** Université Clermont Auvergne, INRAE, UMR 1095 Genetics, Diversity and Ecophysiology of Cereals, Clermont-Ferrand, France

**Keywords:** *Triticum aestivum*, *Fusarium graminearum*, scab, susceptibility factors, S genes, fungal effectors, new resistance sources

## Abstract

Fusarium head blight (FHB), primarily caused by *Fusarium graminearum*, is one of the most devastating fungal wheat diseases. During the past decades, many efforts have been deployed to dissect FHB resistance, investigating both the wheat responses to infection and, more recently, the fungal determinants of pathogenicity. Although no total resistance has been identified so far, they demonstrated that some plant functions and the expression of specific genes are needed to promote FHB. Associated with the increasing list of *F. graminearum* effectors able to divert plant molecular processes, this fact strongly argues for a functional link between susceptibility-related factors and the fate of this disease in wheat. In this review, we gather more recent data concerning the involvement of plant and fungal genes and the functions and mechanisms in the development of FHB susceptibility, and we discuss the possibility to use them to diversify the current sources of FHB resistance.

## Introduction

Fusarium head blight (FHB) is a cereal fungal disease primarily induced by *Fusarium graminearum* (Goswami and Kistler, 2004; Xu and Nicholson, 2009). In wheat, FHB has a direct impact on yield and grain quality, reducing grain weight as well as changing protein accumulation. FHB also causes serious health concerns through the contamination of grains by mycotoxins (e.g., deoxynivalenol, DON, a group 3 carcinogenic toxin), which are resilient to most transformation processes (Li et al., 2014). FHB has become a major threat for wheat crops since the early 1990s, especially in the main producing areas, such as North America, Europe, and China (Zhang et al., 2012). For example, economic losses have been estimated to a total of $1.176 billion over 2015 and 2016 in the United States (Wilson et al., 2018). Such losses are expected to increase as a result of an amplification of the frequency and the intensity of FHB outbreaks due to rises in temperatures and occasional increases in air humidity expected with the climate change (Luck et al., 2011; Shah et al., 2014).

Although the combined use of tolerant wheat cultivars, fungicides, and specific management practices (e.g., tillage and crop rotation) can reduce part of the losses due to the disease (Haidukowski et al., 2005; Hollingsworth et al., 2008; Salgado et al., 2014; Dahl and Wilson, 2018), no efficient strategy can fully control FHB epidemics so far (Mesterházy et al., 2005; Tóth et al., 2008). Primarily addressed through the search for genetic resistance, the last two decades of prolific FHB researches turn out with more than 550 quantitative trait loci (QTLs) (Steiner et al., 2017; Venske et al., 2019), covering the whole genome of wheat but with little effect on resistance improvement and failing in identifying regular resistant genes. Twenty years after the identification of *Fhb1*, the most stable and efficient locus for wheat resistance to FHB (Bai et al., 1999), it was recently shown that a deletion spanning the start codon or an N-terminal mutation of the *TaHRC* gene encoding a putative histidine-rich calcium-binding protein explains part of the *Fhb1*-mediated resistance (Li et al., 2019; Su et al., 2019). Although still in dispute, *TaHRC* constitutes the first susceptibility (*S*) gene to FHB in wheat and directly questions the involvement of susceptibility factors in the disease progress. With this in mind, the purpose of this review is to discuss the growing interest about the determinism of susceptibility to FHB by gathering information from both interacting partners and by emphasizing on its benefits in diversifying the current sources of FHB resistance.

## Fhb Infection Process: Is Susceptibility Behind the Mirror?

Although the recessive nature of some plant resistances has been established for decades, the concept of susceptibility factors, encoded by the so-called susceptibility genes (S genes), has been clearly defined in 2002 by describing the function of the *pmr6* plant gene that promotes the infection process and supports the pathogen’s growth and development (Vogel et al., 2002). Many S genes are now described in plants [reviewed in van Schie and Takken (2014)]. Albeit relatively few, those controlling the wheat/pathogens interactions fit well with this model. For example, the monodehydroascorbate reductase gene, *TaMDHAR4*, has been demonstrated to promote wheat stripe rust infection (Feng et al., 2014). Its mutation results in reducing the hyphae growth of the biotrophic pathogen *Puccinia striiformis*, thus inhibiting its sporulation and enhancing necrosis at the infection site. Further studies in the same interaction evidenced two other S genes, *TaMDAR6* and *TaSTP13* (Abou-Attia et al., 2016; Huai et al., 2020), emphasizing the existence of several S genes in the wheat genome and suggesting that some of them might be implicated in wheat susceptibility-related mechanisms to FHB. If most studies have focused on the genetic determinants of wheat resistance to FHB so far, an interesting alternative is to consider the molecular and the physiological processes that make the host plant susceptible to *F. graminearum*. An extensive literature dealing with large-scale analyses has already shown that, compared to resistant cultivars, the most susceptible ones are characterized by a specific deregulation of genes involved in a wide range of molecular processes (e.g., transcription factors, enzymes involved in primary and secondary metabolism, and defense-related genes), suggesting the intricate participation of a wealth of potential susceptibility factors (Ding et al., 2011; Gottwald et al., 2012; Erayman et al., 2015; Pan et al., 2018; Wang et al., 2018; Brauer et al., 2019).

### Genetics Demonstration of the Existence of Wheat Susceptibility Factors to FHB

**FIGURE 1:**
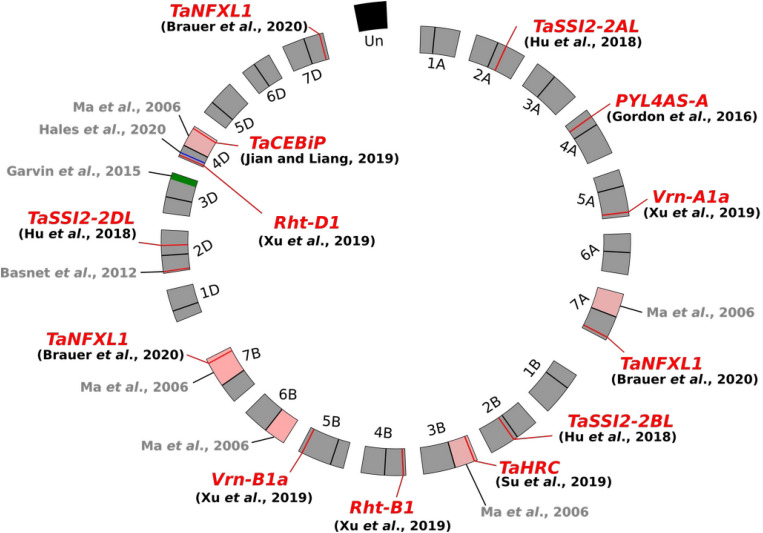
Circos plot of the wheat genome (*Triticum aestivum*) exemplifying Fusarium head blight susceptibility determinants. The three-component genome is represented as a circle including the A, B, and D genomes and their respective chromosomes. The pink areas refer to the deleted chromosome arms in the ditelosomic lines (Ma et al., 2006), the green and blue zones refer to the deleted genomic regions, and the red lines indicate the gene position.

The involvement of putative susceptibility determinants during FHB development has been primarily suggested by studies using wheat aneuploïd lines (Figure 1). Ma et al. (2006) first evidenced that ditelosomic lines lacking in specific chromosome arms displayed an enhanced resistance to *F. graminearum* infection, suggesting the removal of pivotal susceptibility factors along with chromosome fragment deletion. Likewise, Garvin et al. (2015), in an attempt to introgress a new FHB resistance locus from the cultivar (cv.) “*Freedom*” into the susceptible cv. “*Apogee*,” have shown that the most resistant line was characterized by the deletion of a chromosome segment of about 19% of the length of the 3DL arm in comparison with the cv. “*Apogee”* (Figure 1). The wheat line missing this genomic interval resulted in up to 59% decrease of FHB severity as compared to cv. “*Apogee”* and displayed a significant reduction of DON accumulation (Garvin et al., 2015). Similarly, another chromosomal fragment of 31.7 Mbp on the short arm of chromosome 4D was demonstrated to contain potential wheat susceptibility factors to FHB (Hales et al., 2020). Its deletion leads to a significant decrease of *F. graminearum* spreading in wheat spikes. Evidence of susceptibility factors to FHB has also been provided through allele mining studies. The dwarfing allele at the locus *Rht-D1* (*Rht-D1b*, formerly termed *Rht2*) has not been associated to FHB susceptibility by a direct effect of the plant height but rather through a pleiotropic or linkage effect (Draeger et al., 2007). Further experiments demonstrated that, in the cv. “*Spark*,” FHB resistance was largely conferred by the wild allele of the *Rht-D1* gene, while the *Rht-D1b* allele found in the susceptible lines was responsible for approximately 50% of the phenotypic variance associated with the magnitude of initial infection (Srinivasachary et al., 2009). A similar increase of FHB infection has been described for the two particular alleles of the vernalization-related genes *Vrn-A1* and *Vrn-B1* (Xu et al., 2020). An FHB susceptibility source has also been identified in the “*Sumai 3*” *Qfhs.kibr-2DS* QTL (Figure 1), in which a specific allele encoding a multidrug resistance-associated protein was identified in the susceptible “*Sumai 3*”-derived cv. named “*Gamenya*,” unveiling that the FHB susceptibility determinants could be fortuitously inherited from resistant cultivars (Handa et al., 2008; Basnet et al., 2012; Niwa et al., 2014). Such examples suggest that a substantial subset of S genes/factors could be present in the wheat genome and highly conserved among the wheat cultivars. Although most of these studies provide only indirect evidences about the molecular determinism of FHB susceptibility, these results emphasize the relevance of considering the diversity of S genes as a complementary and promising approach to improve wheat resistance to FHB.

### Role of Phytohormones in FHB Development

Several works have already suggested that wheat hormonal pathways play a favorable role in FHB development. For instance, reducing EIN2 expression in wheat, one of the major components of ethylene signaling, decreased the disease symptoms and DON accumulation in the grains (Chen et al., 2009). Further large-scale transcriptomics showed that the ethylene pathway was specifically induced in the FHB-susceptible NAUH117 line, as compared to the resistant Wangshuibai landrace (Xiao et al., 2013). The abscisic acid (ABA) signaling pathway has also been demonstrated to favor *F. graminearum* infection in wheat spikes (Gordon et al., 2016; Wang et al., 2018). A virus-induced gene silencing approach demonstrated a role of the wheat ABA receptor *Ta_PYL4AS_A* (Figure 1), and its close homologs, in mediating FHB susceptibility and in decreasing mycotoxin accumulation (Gordon et al., 2016). Likewise, transcriptional and hormonal profiling showed that wheat genes involved in auxin biosynthesis were highly up-regulated, along with auxin accumulation, in the susceptible cultivars during the *F. graminearum* infection process as compared to the resistant ones (Biselli et al., 2018; Wang et al., 2018; Brauer et al., 2019). Salicylic and jasmonic acids are also widely described for their role in modulating FHB responses and in discriminating resistant *vs*. susceptible cultivars as well (Ding et al., 2011; Gottwald et al., 2012; Sun et al., 2016; Wang et al., 2018). Upon *F. graminearum* infection, their respective actions occur in two phases, an initial induction of salicylic acid happens at the early stages followed by the synthesis of jasmonic acid at the later stages (Ding et al., 2011). In addition, the silencing of the wheat *TaSSI2* gene has been shown to increase FHB resistance, promoting salicylic acid signaling (Hu et al., 2018) and potentially altering the jasmonic acid pathway as demonstrated in *Arabidopsis ssi2* mutant lines (Kachroo et al., 2004). This illustrates further the involvement of these two antagonist hormones in the FHB progress and suggests that susceptibility may involve systemic signals capable of deeply reshaping the plant physiology.

### Shaping FHB Susceptibility in the Course of Grain Development

With a period of maximal susceptibility occurring within 3 days after anthesis (Beccari et al., 2019), FHB develops concomitantly with the grain filling period, during which a large number of plant physiological processes allow a massive accumulation of sugars, lipids, and proteins (Nadaud et al., 2010), resulting in a possible nutrient reservoir in the infection area. Spike ontogeny could thus indirectly and sequentially set up a range of susceptibility factors that can partly explain the dynamics of fungal development during the infection course (Chetouhi et al., 2015, 2016). Extensively boosted during the endosperm expansion, *in planta F. graminearum* growth is associated with massive protein abundance adjustments detectable simultaneously in both plant and fungal proteomes at 48–72 h post-inoculation at anthesis transition (Fabre et al., 2019b). At this stage, extensive metabolic changes in wheat rachis nodes have been reported, including a strong increase of gibberellic acid amount as well as glycolysis intermediates, suggesting that a release of wheat storage carbohydrates could possibly be used for fungal metabolic requirements (Bönnighausen et al., 2018). This is also supported by the converging evidences of large decreases in the expression of genes involved in sucrose and starch metabolism (Erayman et al., 2015; Chetouhi et al., 2016). Starch components, such as amylopectin and amylose, are known to be difficult for the fungus to recycle as a carbon source and for DON production (Oh et al., 2016), unlike sucrose (Jiao et al., 2008; Kawakami et al., 2014), suggesting that the deregulation of the host energy processes at the early stage of the disease could be one of the key factors that determine wheat susceptibility. However, the links are not obvious since many other studies have shown that primary metabolism and especially photosynthesis are extensively rearranged (Long et al., 2015; Biselli et al., 2018; Li et al., 2018; Fabre et al., 2019b). Although this can substantially limit the accumulation of sugars, this could also be seen as a means of constraining the energy requirements necessary to trigger defense mechanisms (Bolton, 2009). In agreement with many previous studies suggesting a key role of chloroplast in plant susceptibility (Lo Presti et al., 2015; Sowden et al., 2018; Han and Kahmann, 2019; Kretschmer et al., 2020), the remodeling of its functioning in wheat spikes during FHB is suspected to be a link between the plant defense responses and the adjustments of primary metabolism. This raises direct questions about the mechanisms used by the fungus to achieve such effects (Fabre et al., 2019b).

## *Fusarium graminearum* Effectors: Knocking at the Wheat Cell Door to Trigger Susceptibility?

Several studies have already demonstrated that plant susceptibility factors could be diverted by a range of pathogen effectors. Consisting of proteins, RNA, and metabolites, effectors are molecules synthesized by the pathogen, delivered in host tissues, and able to alter the structure and the function of the host cell (Hogenhout et al., 2009; Lo Presti et al., 2015). Compared to bacteria, knowledge about fungal effectors remains relatively limited (Niu et al., 2013). However, a number of studies have provided essential information on the ability of *F. graminearum* in interfering with wheat molecular processes. Identifying *F. graminearum* effectors and understanding their roles in the infectious process could be a relevant strategy for identifying wheat susceptibility factors.

### Breaking Wheat’s Defenses

One of the first characterized effectors of *F. graminearum* is the secreted DON mycotoxin (Miller and Young, 1985). Although its synthesis is not necessary for the penetration phase, its role in fungal spreading within the spike has been reported (Bai et al., 2001). DON acts as an inhibitor of protein and nucleotide synthesis in the host cell (Audenaert et al., 2013). Through such an effect, DON is supposed to alter the mitochondrial functions of many eukaryotes, and its role in inhibiting programmed cell death as well as in the expression of defense compounds (chitinases, peroxidases, and pathogen-related proteins) has already been described (Brown et al., 2011; Audenaert et al., 2013; Diamond et al., 2013). A recent report has shown that DON promotes the *TaNFXL1* transcription factor in wheat, leading to FHB susceptibility through uncharacterized mechanisms (Brauer et al., 2020). Other *F. graminearum* effectors have been reported so far, revealing that proteins belonging to the cell-wall-degrading enzymes (CWDEs) are important promoters of wheat susceptibility to FHB (Quarantin et al., 2016, 2019; Paccanaro et al., 2017; Lu and Faris, 2019). For instance, several studies identified *F. graminearum* xylanases with a direct impact on cell wall weakening and an indirect role in enhancing hypersensitive-like symptoms in plant tissues (Paper et al., 2007; Pollet et al., 2009; Sella et al., 2013; Tundo et al., 2015). The FGL1 lipase, another CWDE effector (Voigt et al., 2005), was shown to physically interact with the wheat immunophilin protein FKBP12, altering the establishment of the FKBP12/ERG complex, which finally triggers cell death (Niu et al., 2013). In addition, by degrading the plant cell wall, the FGL1 effector promotes the release of free fatty acids that inhibit the callose deposits associated with the immune responses (Blümke et al., 2014). Similarly, the arabinanase Arb93b, induced during the early stage of FHB, was shown to suppress ROS-activated defense along with its arabinan-degrading activity (Hao et al., 2019). Besides protein effectors, sRNA products have also been reported to control plant responses. The 18-nt-length sRNA (Fg-sRNA1) targets a wheat chitin elicitor-binding protein, which is likely to function in wheat disease resistance signaling pathways (Jian and Liang, 2019). The identification of non-targeted allelic variants could thus guide future research toward “loss-of-susceptibility” forms of resistances.

### Predicted Effector Searches Reveal an Increasingly Complex Arsenal

Although the catalog of characterized *F. graminearum* effectors remains limited, substantial efforts using genomics approaches have provided a large set of new candidates. Using the reference genome sequence, Brown et al. (2012) established a predicted secretome of 574 proteins sharing the structural features of secreted proteins (small size, cysteine-rich proteins, and signal peptides). This revealed a diverse hydrolytic arsenal and a range of putative effectors that could be potentially delivered in the wheat tissues. Secretome was further investigated by focusing on the 190 small secreted cystein-rich proteins (SS): the extracellular localization was confirmed for 25 of them, and the expression of 34 of them was demonstrated as regulated during the FHB progress (Lu and Edwards, 2015). The sequence analysis suggested that 17 SS harbor conserved functional domains such as glycosyl-hydrolase or pathogenesis-related domains, and two of them were homologous to Ecp2, a well-known effector produced by the tomato pathogen *Cladosporium fulvum* (Van den Ackerveken et al., 1993). Other studies dealing with *in vitro* or *in planta* approaches have also been successful in enlarging the list of candidate effectors (Paper et al., 2007; Yang et al., 2012; Ji et al., 2013). By evidencing the protein repertoire found specifically in the extracellular part of the plant tissues or identified in liquid culture and confirmed *in planta* using qRT-PCR, these extended the range of putative function including a number of proteases, esterase, and nucleases. Based on the few structural information available about fungal effectors (Sperschneider et al., 2018), several exploratory reports provided novel insights in their diversity and dynamics (Lysøe et al., 2011; Brown et al., 2017; Fabre et al., 2019a, b). By dissecting the asymptomatic and the symptomatic stages of the FHB infection, Brown et al. (2017) revealed particular gene groups with specific abundance patterns illustrating the early expression of genes involved in the transport of amino acids, in polyamine synthesis and ABC transporters, while hydrolytic carbohydrate-active enzymes and lipases were found at later stages. The delivery of putative effectors by waves at specific stages of the infection has also been confirmed at the protein level using an *in planta* dual-proteome approach (Fabre et al., 2019b). This study further demonstrated that putative effectors could be already accumulated in spores or synthesized within hours, and extensive co-variations were evidenced between abundance changes of effectors and the regulation of plant chloroplast proteins, especially at the beginning of grain cellularization. In addition, strong links were evidenced between the abundance of candidate effectors and strain aggressiveness (Fabre et al., 2019a), emphasizing that increased knowledge of the fungal component could lead to a better understanding of the processes involved in host susceptibility.

## Concluding Remarks

The past decades of researches on FHB in wheat have provided a wealth of information on the genetic and the molecular determinants of the disease progress in spikes, mostly focused on resistance mechanisms. Although still marginally investigated, wheat susceptibility factors to FHB are emerging as key components that determine the fate of the disease, involving a complex molecular dialogue based on the interplay of fungal effectors and their plant targets. Understanding wheat susceptibility still requires many efforts on both partners and needs to fill the gap between wheat and fungal studies. This knowledge will open new strategies in order to control this complex plant/fungus interaction, providing alternative forms of resistance that are potentially more sustainable. While still a challenge, such loss-of-susceptibility forms have already demonstrated their potential to provide efficient and durable sources of disease resistance in crops (Pavan et al., 2010). They represent a promising strategy to control FHB epidemics and may provide a complementary approach to the introgression of gain-of-function resistance genes.

## Author Contributions

FF, FR, and TA organized, prepared, and drafted the manuscript. TL and LB designed, reviewed, and finalized the manuscript.

## Conflict of Interest

The authors declare that the research was conducted in the absence of any commercial or financial relationships that could be construed as a potential conflict of interest.
